# Supplementary material for: Modeling glioblastoma heterogeneity as a dynamic network of cell states
Source: Mol Syst Biol. 2021 Sep 16;17(9):e10105. doi: 10.15252/msb.202010105 (PMC8444284; doi:10.15252/msb.202010105)
Supplement: Supplementary file 5 — Source Data for Figure 3 [file MSB-17-e10105-s001.zip › Figure3A_sourcedata/GSEA_3065/hallmarks_state3.GseaPreranked.1623416274744/HALLMARK_TGF_BETA_SIGNALING.html]

Details for gene set HALLMARK\_TGF\_BETA\_SIGNALING[GSEA]

|  || Dataset | state3 |
| Phenotype | NoPhenotypeAvailable |
| Upregulated in class | na\_neg |
| GeneSet | HALLMARK\_TGF\_BETA\_SIGNALING |
| Enrichment Score (ES) | -0.38065314 |
| Normalized Enrichment Score (NES) | -1.2178102 |
| Nominal p-value | 0.17073171 |
| FDR q-value | 0.28308418 |
| FWER p-Value | 0.992 |
Table: GSEA Results Summary

  

Fig 1: Enrichment plot: HALLMARK\_TGF\_BETA\_SIGNALING      
 Profile of the Running ES Score & Positions of GeneSet Members on the Rank Ordered List

  

| PROBE | GENE SYMBOL | GENE\_TITLE | RANK IN GENE LIST | RANK METRIC SCORE | RUNNING ES | CORE ENRICHMENT || 1 | SPTBN1 |  |  | 61 | 0.394 | 0.0634 | No |
| 2 | TJP1 |  |  | 139 | 0.314 | 0.1108 | No |
| 3 | CTNNB1 |  |  | 373 | 0.224 | 0.1241 | No |
| 4 | HIPK2 |  |  | 545 | 0.193 | 0.1390 | No |
| 5 | SLC20A1 |  |  | 549 | 0.192 | 0.1731 | No |
| 6 | APC |  |  | 616 | 0.184 | 0.1985 | No |
| 7 | ARID4B |  |  | 1269 | 0.117 | 0.1446 | No |
| 8 | TGFBR1 |  |  | 1462 | 0.105 | 0.1413 | No |
| 9 | THBS1 |  |  | 1561 | 0.100 | 0.1480 | No |
| 10 | NCOR2 |  |  | 1781 | 0.087 | 0.1385 | No |
| 11 | BMPR2 |  |  | 2559 | 0.055 | 0.0591 | No |
| 12 | TRIM33 |  |  | 2713 | 0.050 | 0.0504 | No |
| 13 | BMPR1A |  |  | 2736 | 0.049 | 0.0566 | No |
| 14 | SERPINE1 |  |  | 3090 | 0.039 | 0.0230 | No |
| 15 | SKI |  |  | 3493 | 0.028 | -0.0181 | No |
| 16 | SMAD3 |  |  | 3767 | 0.021 | -0.0457 | No |
| 17 | SMURF2 |  |  | 3902 | 0.018 | -0.0580 | No |
| 18 | TGFB1 |  |  | 4110 | 0.012 | -0.0796 | No |
| 19 | XIAP |  |  | 4419 | 0.003 | -0.1143 | No |
| 20 | FNTA |  |  | 4443 | 0.003 | -0.1165 | No |
| 21 | JUNB |  |  | 4467 | 0.002 | -0.1188 | No |
| 22 | CDKN1C |  |  | 4860 | -0.007 | -0.1625 | No |
| 23 | PPP1R15A |  |  | 4983 | -0.010 | -0.1746 | No |
| 24 | SKIL |  |  | 5063 | -0.012 | -0.1815 | No |
| 25 | HDAC1 |  |  | 5401 | -0.021 | -0.2164 | No |
| 26 | KLF10 |  |  | 5514 | -0.025 | -0.2249 | No |
| 27 | SMURF1 |  |  | 5567 | -0.026 | -0.2262 | No |
| 28 | PPM1A |  |  | 5574 | -0.026 | -0.2221 | No |
| 29 | FURIN |  |  | 5987 | -0.039 | -0.2625 | No |
| 30 | BMP2 |  |  | 6331 | -0.050 | -0.2928 | No |
| 31 | CDK9 |  |  | 6358 | -0.051 | -0.2866 | No |
| 32 | SMAD1 |  |  | 6761 | -0.069 | -0.3205 | No |
| 33 | TGIF1 |  |  | 6888 | -0.075 | -0.3215 | No |
| 34 | WWTR1 |  |  | 7187 | -0.093 | -0.3391 | No |
| 35 | MAP3K7 |  |  | 7461 | -0.113 | -0.3502 | Yes |
| 36 | IFNGR2 |  |  | 7647 | -0.132 | -0.3478 | Yes |
| 37 | ID2 |  |  | 7934 | -0.174 | -0.3496 | Yes |
| 38 | ID1 |  |  | 7938 | -0.175 | -0.3187 | Yes |
| 39 | PPP1CA |  |  | 8137 | -0.213 | -0.3032 | Yes |
| 40 | UBE2D3 |  |  | 8174 | -0.221 | -0.2679 | Yes |
| 41 | RHOA |  |  | 8302 | -0.259 | -0.2362 | Yes |
| 42 | ID3 |  |  | 8502 | -0.341 | -0.1980 | Yes |
| 43 | PMEPA1 |  |  | 8520 | -0.346 | -0.1380 | Yes |
| 44 | FKBP1A |  |  | 8601 | -0.404 | -0.0749 | Yes |
| 45 | RAB31 |  |  | 8696 | -0.514 | 0.0063 | Yes |
Table: GSEA details [plain text format]

  

Fig 2: HALLMARK\_TGF\_BETA\_SIGNALING: Random ES distribution      
 Gene set null distribution of ES for **HALLMARK\_TGF\_BETA\_SIGNALING**
